# Preimplantation genetic screening of blastocysts by multiplex qPCR followed by fresh embryo transfer: validation and verification

**DOI:** 10.1186/s13039-015-0140-9

**Published:** 2015-07-08

**Authors:** Yu-Shih Yang, Shun-Ping Chang, Hsin-Fu Chen, Gwo-Chin Ma, Wen-Hsiang Lin, Chi-Fang Lin, Feng-Po Tsai, Cheng-Hsuan Wu, Horng-Der Tsai, Tsung-Hsien Lee, Ming Chen

**Affiliations:** Department of Obstetrics and Gynecology, College of Medicine, National Taiwan University, Taipei, Taiwan; Department of Genomic Medicine, and Center for Medical Genetics, Changhua Christian Hospital, Changhua, Taiwan; Graduate Institute of Medical Genomics and Proteomics, College of Medicine, National Taiwan University, Taipei, Taiwan; Institute of Biochemistry and Biotechnology, Chung Shan Medical University, Taichung, Taiwan; Poyuan Women Clinic, Changhua, Taiwan; Department of Obstetrics and Gynecology, Changhua Christian Hospital, Changhua, Taiwan; Department of Obstetrics and Gynecology, Chung-Shan Medical University, Taichung, Taiwan; Department of Life Sciences, Tunghai University, Taichung, Taiwan

**Keywords:** Aneuploidy, Blastocyst, Fresh embryo transfer, PGS, qPCR

## Abstract

**Background:**

Aneuploidy is an important etiology of implantation failure and quantitative real-time polymerase chain reaction (qPCR) seems a promising preimplantation genetic screening (PGS) technology to detect aneuploidies. This verification study aimed at verifying the impact on reproductive outcomes in *in vitro* fertilization (IVF) cycles using fresh embryo transfer (FET) in which the embryos were selected by blastocyst biopsy with qPCR-based PGS in our settings.

**Results:**

A total of 13 infertile couples with more than once failed in vitro fertilization were enrolled during July to October of 2014. PGS was conducted by qPCR with selectively amplified markers to detect common aneuploidies (chromosomes 13, 18, 21, X, and Y). The design of the qPCR molecular markers adopted the locked nucleic acid (LNA) strategy. The blastocyst biopsy was performed on Day 5/6 and the PGS was done on the same day, which enabled FET. A total of 72 blastocysts were biopsied. Successful diagnoses were established in all embryos and the rate of successful diagnosis was 100 %. The aneuploidy rate was 38.9 % (28/72). 28 embryos were transferred. The clinical pregnancy rate was 61.5 % (8/13) per cycle. Early first trimester abortion was encountered in 1 and the ongoing pregnancy rate was 53.8 % (7/13) per cycle.

**Conclusion:**

This study verified the favorable outcome of adopting PGS with qPCR + FET in our own setting. Expanding the repertoire of aneuploidies being investigated (from a limited set to all 24 chromosomes) is underway and a randomized study by comparing qPCR and other PGS technologies is warranted.

**Electronic supplementary material:**

The online version of this article (doi:10.1186/s13039-015-0140-9) contains supplementary material, which is available to authorized users.

## Background

Preimplantation genetic screening (PGS) by Day 3 cleavage stage embryo biopsy followed by examination with fluorescence *in situ* hybridization (FISH) was once popular strategy for women with advanced maternal age (AMA) [1]. This strategy was based upon an assumption that aneuploidy, which is associate with AMA, is an important factor for implantation failure and therefore such strategy can improve the reproductive outcome [2]. However, a famous randomized study published by Mastenbroek and colleagues reported that such FISH-based PGS with Day 3 biopsy did not confer advantages in women with AMA regarding the live-birth rates [3], and it is then called the “Mastenbroek controversy”. This unexpected finding was subsequently proved by many randomized controlled trials [4] except in one series [5]. Some other researchers thus proposed different strategies including adopting different timing of biopsy (trophectoderm biopsy versus cleavage-stage blastomere biopsy), different molecular technologies which can detect all 24 chromosomes instead of only a few selected chromosomes (which is a major limitation of FISH), and different timing of embryo transfer (fresh or frozen) [1, 6, 7].

Mounting evidences had suggested blastomere biopsy at Day 3 cleavage stage embryos does impair the implantation potential whereas trophectoderm biopsy at Day 5/6 blastocyst does not [8–10]. Meanwhile, it is straightforward that using more sophisticated molecular tools may select a better or “more normal” embryo. Competing technologies include array comparative genomic hybridization (CGH), single nucleotide polymorphism (SNP) array, quantitative real-time polymerase chain reaction (qPCR), and next generation sequencing (NGS) [11]. Amongst them, NGS remains to be mostly investigational because of the resources and cost of bioinformatics analyses [12, 13], and therefore the most feasible tools at the present seem to be qPCR and array-based technologies, including SNP array and array CGH [8, 14–18]. It is now known that mosaicism is a common phenomenon in early human embryo development, and therefore how to select the embryos with best implantation potential becomes a vital question [19]. It is shown by the research groups who proposed for qPCR that qPCR is superior to array CGH since it enjoys a lower false-positive rate for aneuploidy detection (0 % for qPCR and 5.4 % for array CGH in 122 embryos analyzed), and thereby preserving more embryos available for transfer and ensues a better reproductive outcome [14].

There are now commercially available PGS platforms using array CGH in the market (such as those produced by BlueGnome, UK) and has become very popular because of the feasibility of an easy-to-use system, especially for *in vitro* fertilization (IVF) centers without a genetic lab as a supporting resort [11]. However, since the outcomes achieved by the team proposing qPCR are compellingly excellent. It thus poses stress upon researchers of the similar field to verify qPCR as a replacement or an alternative to the current popular PGS by array CGH platforms [14].

In this small verification study, we aimed to assess the PGS by qPCR strategy coupled with trophectoderm biopsy at Day 5/6 blastocyst stage. In the initial stage we first verified qPCR only at selected common aneuploidies including chromosome 13, 18, 21, X, and Y (these aneuploidies can be confirmed by FISH). The reasons we assessed only selected chromosomes are three: First, the detail design and experimental conditions regarding qPCR on all 24 chromosomes are patent protected and we need staged efforts to devise and validate our own. Therefore, in this study we adopted a method called locked nucleic acid (LNA) technology to design our qPCR platform [20]; Second, for a rapid overnight diagnosis which is warranted for fresh embryo transfer (FET), it takes a qPCR machine with a 384-well for only one embryo [18], which may not be feasible in real settings in most genetic labs; Third, we believe the “Mastenbroek controversy” may result not only from lacking an effective genotyping system to screen a “better” embryo, but also from the proven impairment of the implantation potential when biopsy at Day 3 cleavage stage embryos [3, 4, 6]. The team proposing qPCR in one hand arguing qPCR is better than FISH because it can examine all 24 chromosomes but in the other hand claiming despite array CGH should have a better resolution than qPCR whereas implying array CGH is “too sensitive” since it has a more false-positive rate when SNP array is used as a gold standard [14, 16, 18]. We thus are interested to know if it is feasible by using a limited qPCR strategy with examination of only selected common aneuploidies (similar to FISH) coupled with trophectoderm biopsy of Day 5/6 blastocyst stage embryos can still achieve a favorable reproductive outcome. It is ethical since it is by theory better than the trial protocol reported in the “Mastenbroek controversy” trial [3], in which a similar number of aneuploidies was assessed by FISH but the biopsy timing was set at Day 3 (when cleavage-stage embryos), a timing now being recognized to be inferior to Day 5/6 blastocyst stage regarding the implantation potential [9]. After the feasibility is confirmed in our setting, it is then justified to expand the repertoire of aneuploidies to 9 chromosomes (13, 15, 16, 17, 18, 21, 22, X and Y. See Rubio et al., 2013^a^ [5]), or 11 chromosomes (7, 9, 11, 13, 14, 15, 18, 21, 22, X and Y. These are the most commonly found trisomies at abortus. See Wang et al., 2014 [21]) and eventually to 24 chromosomes (1–22, X and Y. See Treff et al., 2012 [1]) in the future.

## Results

### Pre-clinical validation

Pre-clinical validation for the PGS by qPCR was performed on 54 surplus frozen embryos (Additional file 1: Table S1). Fifty-three of these embryos were successfully diagnosed and the successful diagnostic rate was 98.1 % (53/54). Twenty-four embryos were found to carry aneuploidy on tested chromosomes and the rate of aneuploidy was thus 44.4 % (24/53). The only embryo failed to provide a confirmed diagnosis showed poor qPCR signal, possibly due to a low amount of or degraded DNA in the biopsied sample. FISH diagnosis for that embryo was negative for numerical disorders involving chromosome 13, 18, 21, X, and Y. The remaining cells of this biopsied embryo were sent for array CGH and the diagnosis was trisomy 22.

### Clinical verification

A total of 72 embryos at Day 5/6 blastocyst stage from 13 patients were biopsied and assessed, and all (100 %) of them were successfully diagnosed (Table 1 and Additional file 1: Table S1). Twenty-eight (38.9 %) of these embryos were aneuploid, which included 4 monosomy 13, 5 monosomy 21, 7 trisomy 13, 1 trisomy 18, 9 trisomy 21, 1 monosomy 13 + trisomy 18, and 1 monosomy 18 + monosomy 21. The remaining 44 (61.1 %) embryos were euploidy, of which 28 were transferred into the 13 patients in 13 cycles. The average number of the embryos being transferred is 2.15, actually only 1–2 embryos were transferred in all women of the Group B (non-AMA group, n = 10). The only 2 women who were transferred for 4 embryos are both in the Group A (AMA group, n = 3). The transfer procedure follows the medical rule of Taiwan that the upper limit of the number of the embryos being transferred is 4. These transfers achieved 8 pregnancies, and therefore the clinical pregnancy rate was 61.5 % (8/13) per cycle. However, one early abortion was noted and the ongoing pregnancy rate up to the second trimester was 53.8 % (7/13) per transfer cycle. There were no monozygotic twinning and a total of 11 sacs were noted. Three women who were transferred for 2 embryos in the Group B had singleton pregnancies, and two women who were transferred for 2 embryos in the Group B and 2 women who were transferred for 4 embryos in the Group A had dizygotic twin pregnancies (Additional file 1: Table S1). The sustained implantation rate was thus 39.3 % (11/28) per transferred fresh embryo (Table 1). Totally, there are 2 of 3 women in Group A had ongoing pregnancies. Six women in Group B had clinical pregnancies, and 5 of them had sustained ongoing pregnancies. There are no statistical significant differences noted between these two groups regarding aneuploidy (Chi square test, p = 0.094), clinical pregnancy (Fisher's exact test, p = 1), and ongoing pregnancy (Fisher's exact test, p = 1). Array CGH for the only abortion case revealed the karyotype was 46,XY.

**Table 1:** Clinical outcomes of PGS by qPCR with fresh embryo transfer (FET) verification series

	Number	%
Total cycles	13	
Embryos analysed	72	
Embryos diagnosed (%)	72	100.0 %
Embryos with diagnosis failure	0	0
Aneuploid embryos	28	38.9 %
Embryo transferred (ET)	28	
Mean embryos transferred	2.15	
Clinical pregnancies (% cycles)	8	61.5 %
Early miscarriages (<12 weeks)	1	12.5 %
Ongoing pregnancies (% cycles)	7	53.8 %
Embryonic sacs	11	
Embryonic heart tone (FHT)	11	
Sustained implantation rate (% ET)	11/28	39.3 %

## Discussion

The major difficulty of comprehensive chromosome screening (CCS) by qPCR for PGS is to devise a primer set to successfully amplify the molecular markers being selected in a single experiment by optimizing the conditions for melting temperatures and experimental time intervals. In this study we successfully adopted a smart design called LNA methodology [20, 22–24] for qPCR PGS and verified the strategy by achieving a favorable ongoing pregnancy rate of 53.8 %. We first validated the genotyping platform we devised by achieving an almost 100 % successful diagnosis rate (98.1 %), and then verified the whole protocol of qPCR PGS followed by FET. Since the sample size is small (n = 13 cycles), it is not surprising that we failed to observe significant difference regarding the aneuploidy rates between the AMA (n = 3) and the non-AMA group (n = 10) in our patients. Meanwhile, the aneuploidy rate in our series (38.9 %) is apparently much lower than the rates observed by 24-chromosome qPCR or array CGH (more than 60 %), which is obviously due to the fact that we only selected a limited set of common aneuploidies that can ensue live births in humans [14, 15].

It has been a routine practice in our settings that PGS by FISH at Day 3 cleavage-stage embryos since 2005 and it is ethical for us to offer our patients who opted for this alternative protocol a strategy which has an advantage of an established less detrimental effect upon implantation potential by changing the timing of biopsy from Day 3 to Day 5/6 and the same repertoire of common aneuploidies being tested (but simply using qPCR instead of FISH) to enable FET [9]. The reason only trisomy 13, 18, 21, and numerical disorders involving X and Y were tested in our settings is because these are the only aneuploidies that may result in live births in humans. For people who opted for this strategy and came to our clinics, their major concern is more focused at aneuploidies instead of live birth rates. In addition, it is well recognized that despite fewer embryos would be available for transfer if being cultured *in vitro* onto the blastocyst stage when compared with the cleavage-stage embryos, the disadvantage may be offset by the fact that the aneuploidy rate of the blastocysts is much lower than of the cleavage-stage embryos. An observational study had reported that karyotypic evolution did exist in early human embryogenesis, in which some cleavage stage embryos classified as aneuploidy by FISH eventually developed into euploid blastocysts if by SNP array [25]. A recent randomized study even pointed out that FISH-based PGS conferred advantages at women who are in the AMA group, but not in those with repeated implantation failures group, as long as the biopsy was performed at the blastocyst stage [5].

A recent randomized trial (the BEST trial) had demonstrated that qPCR PGS CCS followed by single embryo transfer (SET) can enhance the feasibility of SET by selecting a single euploid embryo with high reproductive potential without compromising the delivery rates when compared with transfer of two unselected embryos, which instead resulted in more multiple pregnancies and the associated complications such as preterm delivery, low birth weight, and NICU (Neonatal Intensive Care Unit) admission [26]. We admit our pilot verification study had suffered from the fact that only common aneuploidies of five chromosome pairs (13, 18, 21, X, and Y) were screened and apparently it is the reason that the implantation rate per embryo in our study (39.3 %) is much lower than that in the BEST trial (69 %) [26]. However, our patients were all informed consented and knew the disadvantage before joining the study, and the limitations of the local setting were clearly disclosed to the patients. The patients were free to choose another PGS protocol by array CGH (Option 2, please refer to Patients and methods section) instead.

## Conclusions

The merit of our work is that this is the first effort from research groups other than the original group who proposed the qPCR CCS (Reproductive Medicine Associates of New Jersey, Morristown, NJ, USA) trying to validate and verify this novel genotyping platform which was developed in-house by ourselves. Despite our results did not have matched controls such as those cycles receiving IVF but without PGS, the ongoing pregnancy rate is similar to a previous PGD series from our setting (53.8 % versus 50 %, please refer to Chang et al., 2013 [27]), indicating this protocol is clinically feasible. Future efforts will be made upon expanding the repertoire of the PGS by qPCR CCS to all 24 chromosomes, as well as carefully-designed randomized controlled trials to compare this genotyping platform with others (including no-screening, array CGH, and NGS).

## Patients and methods

### Design and selection of molecular markers for qPCR PGS

An in-house screening system by dual-color qPCR was developed with specific primers for the targeted loci (situated at chromosome 13, 18, 21, X, and Y) and one internal control locus (situated at chromosome 1, the reason we chose chromosome 1 as control is because it is the least found trisomy in humans, see Wang et al., 2014 [21]). A total of 16 targeted loci and one control locus were selected. For the detailed information about the targeted loci, please refer to Table 2. Specific primers for amplification of each locus were designed by using the software Oligo 6.71 (Molecular Biology Insights, Colorado, USA), and all primer sets are flanked on each side of the exon-intron boundary to avoid possible mRNA interference. The applications of LNA-modified probes are well-established for quantifying levels of gene expression with an advantage of reducing complexity [22, 24], but has not been applied to the detection of chromosomal copy number alterations. Two kinds of LNA-modified probes, labeled with FAM™ and HEX™ (Integrated DNA Technologies, Iowa, USA) at the 5’-end, were used for quantifying the genomic copy numbers of targeted loci and control.

**Table 2:** Summary of the targeted regions for screening of common aneuploidies

Control/Target	Chr.	Location	Gene	UCSC genomic region^a^
Control	1	q25	FAM20B	>chr1:179041176 + 179041348
Target	13	q12	MTMR6	>chr13:25823301-25823552
Target	13	q21.1	PCDH8	>chr13:25823445-25823684
Target	13	q32.2	IPO5	>chr13:53419845-53419995
Target	18	q21	MBD1	>chr18:47800159-47800289
Target	18	q22	ZNF236	>chr18:74592149 + 74592311
Target	18	q23	CTDP1	>chr18:77513630 + 77513793
Target	21	q22.11	URB1	>chr21:33706443-33706522
Target	21	q22.11	TIAM1	>chr21:32582475 + 32582617
Target	21	q22.3	TRAPPC10	>chr21:43279112-43279286
Target	21	q22.3	PRDM15	>chr21:43279123-43279253
Target	Y	p11.3	ZFY	>chrY:2844794 + 2844938
Target	Y	q11	KDM5D	>chrY:21871564-21871720
Target	X	p21.3	ZFX	>chrX:24226424 + 24226508
Target	X	p11.22	HUWE1	>chrX:53573627-53573781
Target	X	q12	EFNB1	>chrX:68060064 + 68060172
Target	X	q25	XPNPEP2	>chrX:128873173 + 128873264

^a^ http://genome.ucsc.edu

### Validation of qPCR in detecting common aneuploidies

**Fig. 1:**
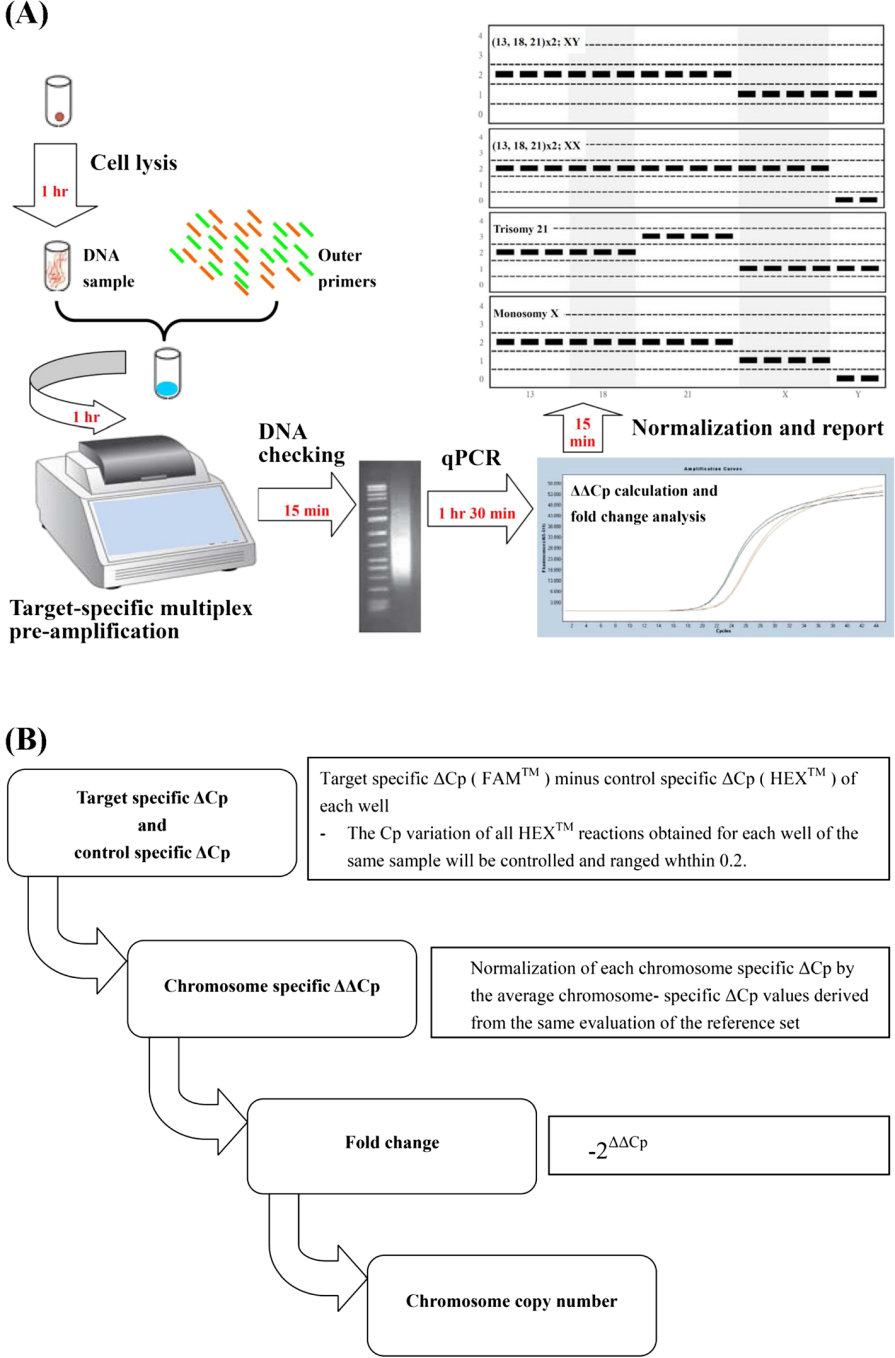
The diagram of the in-house qPCR PGS system. The flowchart of (**a**) detection of common aneuploidies, and (**b**) signal normalization and data analysis

Each 5 cells separated from the cell lines of known common aneuploidies (including trisomy 21, trisomy 13, trisomy 18, 47,XXY, and 45,X) were processed by cell lysis of proteinase K, and the products were subjected to a 50-μL reaction volume of multiplex nested-PCR amplication for 18 cycles using an Applied Biosystems Veriti thermal cycler (Life Technologies, California, USA), and then the PCR products were purified using Agencourt AMPure XP system (Beckman Coulter, California, USA). Dual color hydrolysis probe assays were performed in triplicate to normalize and simpify calculation and to evaluate chromosomal copies, using Lightcycler 480 probes Master (Roche, Mannheim, Germany), a 20-μL reaction volume, a 96-well plate, and a 7 Light Cycler 480 Real-Time PCR System, as recommended by the supplier (Roche, Mannheim, Germany). Each well contains a particular target, and a common control reaction. A unique method of the standard delta delta threshold cycle (ΔΔCp) method was used for relative quantification. In our experiments, the Cp variation of all HEX™ reactions obtained for each well of the same sample will be controlled and ranged in less than 0.2, indicating the test sample was evenly distributed to each well. Each chromosome-specific ΔCp was calculated from the Cp of the FAM™ reactions targeting a specific chromosome minus the control Cp of the HEX™ reactions targeting the chromosome 1 within the same well. The same process was applied to individually determine the ΔCp for each targeted chromosome of the test sample, including reference set of normal male cell lines [BCRC number: 08C0011, 08C0012, 08C0013, 08C0021 and 08C0025]. Each chromosome- specific ΔCp was then normalized to the average chromosome-specific ΔCp values derived from the same evaluation of the reference set, which had been confirmed by FISH method. The calibrated chromosome-specific ΔCp values were used to calculate fold change by considering the ΔΔCp values as the negative exponent of 2, as previously described [18, 23]. The methodology was designed to specifically identify whole-chromosome but not segmental aneuploidy. The flowchart and diagram of the in-house qPCR PGS system were illustrated in Fig. 1. This qPCR was capable of accurate aneuploidy screening in 4 h, which allowed rapid evaluation of the trophectoderm biopsies and therefore provided a feasible opportunity for subsequent FET.

### Pre-clinical validation upon surplus frozen embryos

Fifty-four thawed frozen embryos at blastocyst stage were biopsied and sent for qPCR and FISH analyses. These embryos were retrieved from the surplus frozen embryos of couples who already conceived and would be discarded if not being investigated for research purpose. In those embryos diagnosed with common chromosome aneuploidies, array CGH was used to confirm the diagnoses.

### Clinical verification for fresh embryos

In our setting we used to offer two PGS protocols. One option (Option 1) is Day 3 biopsy followed by PGS with FISH and FET; the other option (Option 2) is Day 5/6 trophectoderm biopsy followed by PGS with array CGH and frozen embryo transfer (because array CGH takes time and FET was not feasible at the study period). Only patients who had history of failed IVF (without PGS) for at least once and who opted for Option 1 were given the chance of joining this study as an alternative. All patients chose to join this study were informed consented and their autonomy was fully respected. They could choose to withdraw from the study at any time during the study period and were fully aware of the alternatives, including sticking to Option 1 or instead chose Option 2 without joining the study. During July to October of 2014, 13 infertile couples were enrolled. Among these 13 patients, 3 of them had AMA (37, 43, 45 years old respectively). The mean age of the total 13 patients was 34.1 years. No confounding factors that may affect implantation such as immune aberrations (antiphospholipid antibody syndrome in the mother), balanced translocation carriers (in both couples), and thrombophilias (protein C/S/antithrombin III deficiency) existed in these couples when they were enrolled. Clinical pregnancy was defined as positive urine HCG. Ongoing pregnancy rate (per cycle) was defined as those pregnancies proceeding into second trimester (and for each embryo being transferred, sustained implantation rate was used). 13 couples were classified as those whose age is older or equal to 35 years (Group A), and those whose age is less than 35 years (Group B). Chi square test or Fisher’s exact test was used to compare the reproductive outcomes between the groups regarding the rate of aneuploidy, clinical pregnancy, and ongoing pregnancy. Notably according to the regulations of Taiwan government, the patients would not know the results of the fetal sex. Even in aneuploidies involving sex chromosomes (specifically 47,XXY and 45,X), the results would not be disclosed to patients and only “aneuploidy” would be told to the patients and the aneuploid embryos would not be selected for transfer. In those embryos diagnosed to have chromosomal aneuploidies by qPCR, array CGH was used to confirm the diagnoses.

## Additional file

Additional file 1: Table S1. Summary of the pre-clinical validation (for 54 surplus frozen embryos) and clinical verification (for 13 patients with 54 embryos) of PGS by qPCR.

## Abbreviations

AMA: Advanced maternal age
CCS: Comprehensive chromosome screening
CGH: Comparative genomic hybridization
FET: Fresh embryo transfer
FISH: Fluorescence *in situ* hybridization
IVF: *in vitro* fertilization
LNA: Locked nucleic acid
NGS: Next generation sequencing
PGS: Preimplantation genetic screening
qPCR: Quantitative real-time polymerase chain reaction
SET: Single embryo transfer
SNP: Single nucleotide polymorphism
