# Supplementary material for: Preimplantation genetic screening of blastocysts by multiplex qPCR followed by fresh embryo transfer: validation and verification
Source: Mol Cytogenet. 2015 Jul 8;8:49. doi: 10.1186/s13039-015-0140-9 (PMC4495615; doi:10.1186/s13039-015-0140-9)
Supplement: Additional file 1: Table S1. — Summary of the pre-clinical validation (for 54 surplus frozen embryos) and clinical verification (for 13 patients with 54 embryos) of PGS by qPCR. [file 13039_2015_140_MOESM1_ESM.docx]

Table S1. Summary of the pre-clinical validation (for 54 surplus frozen embryos) and clinical verification (for 13 patients with 54 embryos) of PGS by qPCR
